# Spermine and Spermidine Priming against *Botrytis cinerea* Modulates ROS Dynamics and Metabolism in Arabidopsis

**DOI:** 10.3390/biom11020223

**Published:** 2021-02-05

**Authors:** Henry Christopher Janse van Rensburg, Anis M. Limami, Wim Van den Ende

**Affiliations:** 1 Laboratory of Molecular Plant Biology, KU Leuven, Kasteelpark Arenberg 31, 3001 Leuven, Belgium; henry.jansevanrensburg@kuleuven.be; 2 Univ Angers, Institut Agro, INRAE, IRHS, SFR QUASAV, F-49000 Angers, France; anis.limami@univ-angers.fr

**Keywords:** polyamines, spermine, spermidine, *B. cinerea*, priming, Arabidopsis, sugars, reactive oxygen species, amino acids

## Abstract

Polyamines (PAs) are ubiquitous small aliphatic polycations important for growth, development, and environmental stress responses in plants. Here, we demonstrate that exogenous application of spermine (Spm) and spermidine (Spd) induced cell death at high concentrations, but primed resistance against the necrotrophic fungus *Botrytis cinerea* in Arabidopsis. At low concentrations, Spm was more effective than Spd. Treatments with higher exogenous Spd and Spm concentrations resulted in a biphasic endogenous PA accumulation. Exogenous Spm induced the accumulation of H_2_O_2_ after treatment but also after infection with *B. cinerea*. Both Spm and Spd induced the activities of catalase, ascorbate peroxidase, and guaiacol peroxidase after treatment but also after infection with *B. cinerea*. The soluble sugars glucose, fructose, and sucrose accumulated after treatment with high concentrations of PAs, whereas only Spm induced sugar accumulation after infection. Total and active nitrate reductase (NR) activities were inhibited by Spm treatment, whereas Spd inhibited active NR at low concentrations but promoted active NR at high concentrations. Finally, γaminobutyric acid accumulated after treatment and infection in plants treated with high concentrations of Spm. Phenylalanine and asparagine also accumulated after infection in plants treated with a high concentration of Spm. Our data illustrate that Spm and Spd are effective in priming resistance against *B. cinerea*, opening the door for the development of sustainable alternatives for chemical pesticides.

## 1. Introduction

Polyamines (PAs) are small aliphatic polycations that are found in all eukaryotes. In plants, the major PAs include the diamine putrescine (Put), the triamine spermidine (Spd), and the tetraamine spermine (Spm). They occur in free and conjugated forms as their positive charge allow them to interact with polyanionic molecules such as phospholipids, proteins, and nucleic acids. PAs have diverse functions in several physiological and developmental responses such as cell division, embryogenesis, and senescence [1,2,3]. The regulation of PA metabolism, conjugation, and localization are thus important to maintain cellular homeostasis. However, PAs also play important physiological roles during (a)biotic stress responses and biotic/abiotic stress cross-tolerance in plants [4,5,6]. As such, PA biosynthesis and degradation are highly regulated by environmental stimuli [7].

The accumulation of PAs during (a)biotic stresses modulates plant defences [8,9,10]. PA-related gene expression significantly changes in response to biotic interactions, with major fluctuations in their concentration and localization [11]. Since both plants and pathogens are able to synthesize PAs, it is challenging to dissect their origin during their interaction. There are numerous results of pathogens taking control of plant metabolism, including PA metabolism, through the use of effectors [12,13]. Interestingly, PA metabolism is also heavily affected by beneficial microorganisms [14], suggesting that it is probably a combination of plant- and microorganism-induced changes.

One of the earliest reports indicating fluctuations in PA levels during pathogen infection showed that Spd accumulated in barley infected with *Puccinia hordei*, a biotrophic fungus [15]. Later, it was shown that PA accumulation is also stimulated upon fungal elicitor treatment [16], suggesting that the metabolism of PAs form part of general defence strategies. The accumulation of PAs during biotic interaction is mainly attributed to the induction of the biosynthetic enzymes arginine decarboxylase (ADC) and ornithine decarboxylase (ODC) [11]. Endogenous accumulation of PAs is typically associated with resistance against biotrophic pathogens [5], but with increased susceptibility to necrotrophs [17]. In particular, the apoplastic metabolism of PAs appears to be differentially regulated in response to biotrophic and necrotrophic pathogens. Increased PA accumulation (both total and apoplastic) during necrotrophic infection with *Sclerotinia sclerotiorum* in transgenic lines enhanced necrotic lesion development [17]. It is believed that PA oxidation by PA oxidase (PAO) in the apoplast contributes to cell death, partially through the production of H_2_O_2_. On the other hand, this H_2_O_2_ may also be associated with wound healing [18] and reinforcement of the cell wall during infection of the necrotrophic fungus *Ascochyta rabiei* in chickpea [19]. Plants over-accumulating Spd were found to be more susceptible to the necrotrophic fungus *Botrytis cinerea* whereas they were similar to wild-type plants in response to the necrotroph *Alternaria solanii* [20]. On the other hand, Spm induces resistance against *B. cinerea* by activating systemic acquired resistance (SAR) [20]. Leaf disks of *Solanum lycopersicum*, *Phaseolus vulgaris*, and Arabidopsis pre-incubated with Spm and immediately infected were less susceptible to *B. cinerea* infection [21]. It was proposed that Spm functions as a signalling molecule acting in plant defence responses and senescence [22,23,24]. In fact, a potential polyamine sensor has been identified in bacteria [25]. Recently, Spm was linked to the accumulation of the signalling lipid phosphatidic acid in the roots of seedlings and mature leaves of Arabidopsis [26]. It was demonstrated that Spm was more effective at low concentrations than Spd. Furthermore, PA uptake in the cells was required for a successful response. In conclusion, data regarding the role of PAs during plant stress responses, towards necrotrophic pathogens in particular, are complex and not only depend on the pathogen and plant species but most importantly also on the exact timing and location of PA production. Clearly, we are only at the beginning of our understanding on the complex crosstalk between PA, H_2_O_2_, and lipid signalling pathways in plants.

Besides the direct effects employed by PAs and their metabolism during plant–pathogen interaction, they can also affect alternative stress response pathways and components. One of the breakdown products of PA oxidation is the non-proteinogenic amino acid γ-aminobutyric acid (GABA). GABA is known to alleviate several abiotic and biotic stresses in plants, and is also believed to function as a signalling molecule [27]. Increased PA catabolism towards GABA has been shown to contribute to resistance against necrotrophic (*B. cinerea*) and saprophytic (*Aspergillus flavus*) fungi [10,28]. Grapevine plants exposed to drought showed higher catabolism of PAs and subsequent GABA accumulation, which improved resistance against *B. cinerea* [28]. In Arabidopsis, exogenous application of Put, Spd, and Spm induced the activities of PAOs to promote polyamine breakdown [29], as well as the pathway responsible for GABA synthesis. It seems possible that PA accumulation associates with temporal GABA fluctuations, in turn promoting plant defence pathways through priming [30]. However, apoplastic polyamine oxidation can also produce H_2_O_2_, which can be beneficial towards necrotrophic pathogens at their later infection stages. This further emphasizes the temporal importance of PAs and their breakdown.

Further, there seems to be a link between PA metabolism and sugar metabolism and signalling in plants. Exogenous sucrose (Suc) application induced the total accumulation of Put, Spd, and Spm in Arabidopsis seedlings during chemical stress [31]. It was proposed that the crosstalk between Suc signalling and PAs may be responsible for the prevention of programmed cell death and increased antioxidant activities. Although the interplay of PAs with sugar signalling pathways remains largely unexplored in plants, PA metabolism seems to be interlinked with carbon (C) flux. In growing seedlings (sinks), it was suggested that the redirection of C and nitrogen (N) metabolism in target of rapamycin (TOR)-inhibited plants might be attributed to PAs [32], as PAs were previously linked to anabolic processes and C:N signalling [33,34]. Transgenic plants accumulating PAs show major shifts in their C and N metabolism and tend to promote catabolic processes. This suggests that PAs are intricately linked with the energy/sugar status of plants and that their accumulation under stress may be associated with (or preceded by) sugar signalling events. The ability to PAs to regulate the C:N ratio is further supported by studies indicating that PAs can regulate the activity of nitrate reductase (NR) by affecting its accumulation and activation state in plants [35,36]. Nitrate reductase is an important enzyme to provide N for amino acid synthesis in plants, and its activity is sensitive to several (a)biotic stresses [37,38].

Even though a role for PA accumulation during several biotic stresses was proposed, research on the ability of PAs to act as priming agents against necrotrophic pathogens is still in its infant stage. With controversial findings on the effect of PA accumulation during necrotrophic infection and reports of enhanced susceptibility in plants over-accumulating PAs, here, we explored, contrary to previously [21], a scenario where PA pre-treatment and infection were temporally separated by 3 days, providing evidence in favour of real priming, leading to enhanced resistance against infection with the necrotrophic fungus *B. cinerea*. We also looked at the role of exogenous PAs on endogenous PA contents, sugar and amino acid levels, reactive oxygen species (ROS) dynamics, and ROS scavenging enzymes after treatment and infection. We also focused on NR activity to better understand the dynamic interplay of PAs with plant C and N metabolic processes.

## 2. Materials and Methods

### 2.1. Plant Growth Conditions

*Arabidopsis thaliana* (Col-0) seeds were stratified for 3 days at 4 °C in the dark before being transferred to square pots (9 × 9 × 8 cm), 5 plants per pot, in a mixture of potting soil and vermiculite (3:2). Plants were grown under a 12 h light (21 °C) and 12 h dark (18 °C) light cycle under cool-white fluorescent lamps with 100 μmol·m^−2^·s^−1^ light intensity and 60% relative humidity in a Conviron (Berlin, Germany) growth chamber.

### 2.2. Plant Treatments

Plants of 4–5 weeks old were randomly divided into the different treatment groups, and well-watered 1 h before treatments. All priming solutions were prepared in double-distilled (dd) H_2_O containing 0.0001% Tween-20 (Acros Organics, Morris Plains, NJ, USA) as surfactant. Plants were treated by spraying the leaves of plants with Spd or Spm (Acros Organics, Morris Plains, NJ, USA) at different concentrations and a control (ddH_2_O containing 0.0001% Tween-20) treatment by using a spraying bottle. Each pot was sprayed at a distance of +/−15 cm with 5 mL solution to completely cover the surface of the rosette leaves. Plants were then randomly divided and returned to the growth chamber.

### 2.3. B. cinerea Cultivation and Infection

For all experiments, *B. cinerea* strain B05.10 obtained from Prof. Dr. Barbara De Coninck (KU Leuven, Division of Crop Biotechnics, Leuven, Belgium) were used [39,40]. To generate spores, we grew *B. cinerea* on 24 g/L potato dextrose agar (PDA) for 14 days at 21 °C in the dark. Spores were harvested using ddH_2_O containing 0.0001% Tween-20 by gently scraping the surface using a 1 mL pipette tip. Spore suspension was then filtered through glass wool to remove mycelium. Concentration was determined using a Neubauer haemocytometer (Fison Scientific Equipments, Loughborough, UK) and adjusted to 1 × 10^5^ spores per mL in sterile 12 g/L potato dextrose medium. Infection control (IC) contained 12 g/L potato dextrose medium with no spores. Spores were incubated at room temperature for 4 h prior to infection to allow for synchronous germination.

Infections were conducted as described previously [40]. Three source leaves per plant (rosette leaves 5–7) were cut, rinsed in ddH_2_O, and blotted with paper towel to remove residual priming compounds from the leaves. Control treatments were handled alongside to ensure consistent handling between controls and treatments. Leaves were then placed adaxial side upwards in a square Petri dish (Greiner Bio-One, Frickenhausen, Germany) lined with moist paper towel to maintain high humidity. Leaves were subsequently infected by pipetting individual droplets of 5 µL of either the infection buffer or IC buffer to the tip of each leaf. Plates were then sealed with parafilm and placed in an infection room maintained at 18 °C with 12 h day and 12 h night cycle. Infections were allowed to continue for 72 h for lesions to develop. Disease scoring was performed by taking photos of lesions 72 h after infection and the area determined using a reference and the software ImageJ 1.5T (National Institute of Health, Bethesda, MD, USA, https://imagej.nih.gov/ij/ accessed on 5 February 2021).

### 2.4. Polyamine Extraction and Quantification by GC–MS

Gas chromatography coupled with mass spectrometry was carried out with a 436-GC coupled to a Simple Quadruple (SQ) SCION MS (Bruker, Durham, UK). The column was an RTX-5 w/integra-Guard (30 m × 0.25 mm i.d. + 10-m integrated guard column; Restek, Evry, France). Leaf samples (15 mg of powder from freeze-dried material) were ground in a mortar in liquid N_2_ and then in 2 mL of 80% methanol in which ribitol (100 μmol L^−1^) was added as an internal standard. Extracts were transferred to 2 mL Eppendorf tubes and centrifuged at 12,000× *g* (4 °C) for 25 min. Supernatants were SpeedVac-dried and stored at −80 °C until analysis. Methoxyamine was dissolved in pyridine at 20 mg mL^−1^, and 50 μL of this mixture was used to dissolve the dry individual samples and polyamine standards (putrescine, spermidine, and spermine). Following vigorous mixing, samples were incubated for 90 min at 30 °C with shaking. A total of 50 μL of *N*-methyl-*N*-(trimethyl-silyl)trifluoroacetamide was then added, and the mixture was vortexed and incubated for 30 min at 37 °C with shaking. Before loading into the gas chromatography autosampler, a mix of a series of 12 alkanes (chain lengths of C_10_–C_34_) was included. Analyses were performed by injecting 1 μL in splitless mode at 280 °C injector temperature. The chromatographic separation was performed in helium as a carrier gas at 1 mL min^−1^ in the constant flow mode and using a temperature ramp ranging from 70 °C to 320 °C between 4 and 22 min, followed by 5 min at 320 °C. Ionisation was made by electron impact at 70 eV, and the mass spectra acquisition rate was 20 spectra s^−1^ over the *m/z* range 70 to 500. Peak identity was established by comparison of the fragmentation pattern with mass spectra of polyamine standards and available databases (National Institute of Standards and Technology, Gaithersburg, MD, USA), and by retention index using the alkane series as retention standards. The integration of peaks was performed using the Bruker MS Workstation software.

### 2.5. H_2_O_2_ Extraction and Quantification

Extraction and quantification of H_2_O_2_ was carried out with the eFOX method as explained previously [41]. Leaves 5–7 (similar to the ones used for infection) were harvested and frozen in liquid nitrogen, whereafter immediate extraction occurred to prevent loss of H_2_O_2_ during storage. Samples were grinded in ice-cold 5% trichloroacetic acid (TCA) using a pre-cooled mortar and pestle. Extracts were then centrifuged for 10 min at 15,000× *g* (4 °C) to remove plant debris and extract added to eFOX reagent in a 1:1 ratio. The eFOX reagent consisted of 500 µM ferrous ammonium sulphate (Honeywell-Fluka, Morris Plains, NJ, USA), 200 mM sorbitol, 200 µM xylenol orange (Honeywell-Fluka, Morris Plains, NJ, USA), and 1% ethanol prepared at double the final concentration in 50 mM H_2_SO_4_. Reactions were incubated at room temperature for 30 min, and the optical density (OD) was measured at 550 nm and 800 nm using a spectrophotometer (Spectronic Genesys 5, Thermo Scientific, Waltham, MA, USA). The concentration of H_2_O_2_ was calculated by using a standard curve prepared from 30% H_2_O_2_ (Sigma-Aldrich, St. Louis, MO, USA) in a range between 0 and 200 µM, similar to the sample preparation.

### 2.6. Antioxidant Enzyme Extraction and Activity Measurements

For antioxidant enzyme analysis, catalase (CAT), ascorbate peroxidase (APX), and guaiacol peroxidase (GPX) enzymes were extracted as previously described [42]. Rosette leaves (5–7 in number) were grinded in liquid nitrogen and 100 mg was extracted with 300 µL extraction buffer (100 mM phosphate buffer (pH 7.0), 0.1% Triton X-100, 15% glycerol, 1 mM phenylmethylsulfonyl fluoride (PMSF), 1 mM ascorbic acid, and 0.35 mM β-mercaptoethanol) by grinding with a plastic micro pestle inside a 1.5 mL Eppendorf tube for 30 s. Samples were then centrifuged for 10 min at 15,000× *g* (4 °C) to remove plant debris. Extracts were kept at −80 °C until further analysis, except APX, which was measured immediately.

CAT activity was measured as previously with minor modifications [42,43]. The breakdown of H_2_O_2_ was measured at an OD of 240 nm. Activity was measured in 2 mL 100 mM phosphate buffer (pH 7.0) in a quartz glass cuvette by adding 40 µL enzyme extract. Background was measured for 30 s before the addition of 40 µL 1 M H_2_O_2_ to start the reaction. The decrease in OD_240_ was measured for 5 min at 10 s intervals on a Spectronic Genesys 5 spectrophotometer (Thermo Scientific, Waltham, MA, USA). Activity was calculated from the linear range of the reaction. Blanks consisted of a general control (buffer and H_2_O_2_) and sample control (buffer and enzyme extract).

APX was assayed as described previously [44,45]. Activity was measured as the oxidation of ascorbic acid from enzymes extracted in the presence of 1 mM ascorbic acid. The oxidation of ascorbic acid was measured as the decrease in absorbance at a wavelength of 290 nm. Reactions were carried out in a volume of 2 mL consisting of 1.870 mL of 100 mM phosphate buffer (pH 7.0) containing 0.5 mM ascorbic acid and 30 µL enzyme extract. Background was measured for 1 min before the addition of 100 µL of 27 mM H_2_O_2_ to start the reaction. Reactions were followed for 5 min at 10 s intervals. An additional blank consisted of reaction mixture and H_2_O_2_ without the addition of enzyme. Activity was determined in the linear range.

GPX activity was measured calorimetrically using guaiacol as the substrate, as explained previously [46]. Extracted enzyme (25 µL) was added to a reaction mixture consisting of 1.875 mL 100 mM phosphate buffer (pH 7.0) and 1 mL of 25 mM guaiacol in a glass quartz cuvette. Background was measured for 1 min before the reaction was started by adding 100 µL of 2% H_2_O_2_. The change in absorbance was measured spectrophotometrically at an OD of 480 nm for 5 min at 10 s intervals. A second blank contained H_2_O_2_ with no enzyme.

For all enzyme reactions, the activity was calculated from the linear range of the reaction, with enzyme activity expressed as units (U)·mg protein^−1^, where 1 U is equal to the change in OD of 0.01 per min.

### 2.7. Soluble Sugar Extraction and Quantification

Soluble sugars were extracted and quantified as previously explained [40]. Frozen leaf material was grinded in liquid nitrogen using a mortar and pestle, and 100 mg extracted in 1 mL ddH_2_O by boiling for 15 min at 95 °C. Samples were vortexed and centrifuged for 10 min at 15,000× *g* to remove plant debris and supernatant was desalted by applying 200 µL to a Dowex anion and cation exchange column prepared in glass Pasteur pipettes. Columns were washed 6 times with 200 µL ddH_2_O and the flow through diluted 1:1 in 20 µM rhamnose H_2_O serving as internal standard. Samples were quantified by injecting 6 µL on a High performance anion exchange chromatography (HPAEC) with pulsed amperometric detection (PAD) Dionex 5000 (Thermo Scientific, Whaltam, MA, USA) with separation on a CarboPac PA100 column (Thermo Scientific, Whaltam, MA, USA) and a mobile phase of 90 mM NaOH, as described previously [47]. Concentrations were calculated using standards of 10 µM of each sugar running alongside.

### 2.8. Enzyme Extraction and Nitrate Reductase Activity Measurement

Nitrate reductase activity was measured as previously explained with minor modifications [48]. Previously harvested leaves were grinded in liquid nitrogen and 100 mg material was extracted with 400 µL extraction buffer (50 mM 4-(2-hydroxyethyl)-1-piperazineethanesulfonic acid (HEPES)-KOH (pH 7.5), 10% (*v*/*v*) glycerol, 0.1% (*v*/*v*) Triton X-100, 10 mM MgCl_2_, 1 mM Ethylenediaminetetraacetic acid (EDTA), 1 mM benzamidine, 1 mM ɛ-aminocapronic acid, 1 mM PMSF, 1× cOmplete Protease Inhibitor Cocktail (Roche, Diagnostics Ltd., Mannheim, Germany), 1 mM Dithiothreitol (DTT), and 20 µM flavin adenine dinucleotide) by vortexing for 30 s. Samples were subsequently centrifuged for 5 min (4 °C) at 13,000× *g*, and the supernatant was used in reactions. Assays were performed by the addition of 100 µL extract to 450 µL assay buffers. Assay buffers consisted of 50 mM HEPES-KOH (pH 7.5), 0.04% Triton X-100, 10 µM Na_2_MoO_4_, 0.5 mM DTT, and 20 mM KNO_3_, with the addition of 10 mM MgCl_2_ for the selective NR (active) reaction and 5 mM EDTA for the total NR reaction. Reaction mixture containing enzyme was incubated for 2 min at 25 °C before the reaction was started by adding 50 µL of 3 mM reduced nicotinamide adenine dinucleotide (NADH). After 10 min, the reactions were terminated by adding 50 µL of 0.6 mM zinc acetate. For blanks, zinc acetate was added before the addition of enzyme. Subsequently, unreacted NADH was removed by adding 75 µL of phenyl methosulphate to the reactions and by incubating for 15 min in the dark. Finally, 300 µL of 1% sulphanilamide in 3 N HCL and 300 µL of 0.02% *N*-(1-napthyl)-ethylenediamine were added and incubated for 20 min for colour development. Samples were then centrifuged for 5 min at 14,000× *g* to remove zinc acetate and the azo-dye formed measured at an OD of 540 nm using a Multiskan Ascent 96/384 Plate Reader (Thermo Scientific, Waltham, MA, USA). The concentrations of nitrite formed per reaction were interpolated from a standard curve prepared between 0 and 200 µM using NaNO_2_.

### 2.9. Amino Acid Extraction and Quantification

Amino acids were extracted by adding 1 mL ddH_2_O to 100 mg ground leaf material and boiling at 95 °C for 15 min. Samples were centrifuged at 15,000× *g* (4 °C) for 10 min before 25 µL of supernatant was diluted in 25 µL Nor-Valine (internal standard). Amino acids were quantified by reverse-phase high-performance liquid chromatography (HPLC) (Shimadzu, Kyoto, Japan) by derivatisation with O-phtalaldehyde before injection. Separation was carried out using an YMC-Triart C18 column and mobile phases consisting of buffer A (50 mM KH_2_PO_4_ (pH 6.5), 0.7% *v*/*v* tetrahydrofurane) and buffer B (acetonitrile/methanol/water 45:40:15) using the following gradient: 96% A and 4% B from 0 to 6 min; 92% A and 8% B from 6 to 18 min; 85% A and 15% B from 18 to 32 min; 67% A and 33% B from 32 to 50 min; 100% B from 50 to 53 min. Amino acids were detected using a fluorescence detector at λex = 230 nm and λem = 450 nm.

### 2.10. Experimental Setup

**Figure 1:**
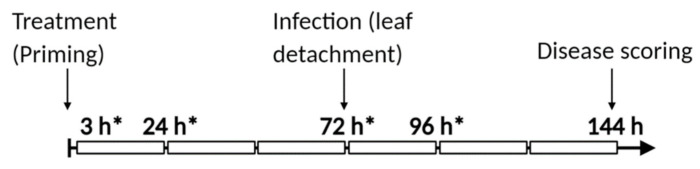
Schematic representation of the experimental setup. At 0 h, plants were treated by spraying with the different treatments, followed by *Botrytis cinerea* infection 72 h later. Disease analysis was conducted 72 h after infection (144 h after treatment). At 3, 24, and 72 h (time of infection) after treatment and 24 h after infection (96 h after treatment), samples were harvested for analysis. All samples used for analysis were from the source leaves (rosette leaves 5–7) of 4–5-week-old Arabidopsis plants. * indicate sample harvesting times.

The experimental setup is displayed in Figure 1. Figure S1 illustrates an example of lesions developed 72 h after infecting detached leaves.

### 2.11. Graphical Preparation and Statistical Analysis

Graphs were prepared using GraphPad Prism 8.0.0 and Inkscape 1.0. Statistical analysis was performed using GraphPad Prism version 8.0.0 for Windows (GraphPad Software, San Diego, CA, USA, www.graphpad.com accessed on 5 February 2021). For disease scoring, statistical significance was determined by one-way ANOVA and Dunnett’s multiple comparisons test and adjusted *p*-values. For all other analyses, statistical significance between treatments was determined by two-way ANOVA followed by Tukey’s multiple comparison and adjusted *p*-values.

## 3. Results

### 3.1. High Concentrations of Polyamines Induce Cell Death in Arabidopsis Source Leaves

In a previous study, exogenous application of PAs induced necrotic lesions in tobacco plants at a concentration of 10 mM [49]. Similarly, plants accumulating high concentrations of PAs, or plants infiltrated with PAs showed more severe lesion development when challenged with necrotrophic pathogens [17,20]. Considering that a high concentration of PAs in the apoplast leads to excessive H_2_O_2_ accumulation through PA oxidation, promoting cell death, exogenous treatments could induce a similar phenomenon. Exogenous PAs also induce the accumulation of phosphatidic acid, known to induce cell death in Arabidopsis [50]. However, treatments at lower concentrations might only induce minor accumulation of H_2_O_2_, which can be effective to induce plant defences against subsequent necrotrophic pathogens [51,52]. In our pilot experiments, we also observed the development of cell death at higher concentrations, and thus we set out to identify the highest concentration of Spm and Spd that could be used without inducing visible lesions (Table 1).

Arabidopsis plants were sprayed with different concentrations of Spd and Spm and monitored for the number of leaves per plant showing visible lesion development over 120 h (Table 1). Images of plants treated with different concentrations (100 µM, 500 µM, and 1 mM) of Spd and Spm after 120 h can be found in Figure S2. Plants treated with 1 mM Spm showed visible lesions already after 48 h (Table 1), specifically on the mature source leaves (Figure S2). For Spd at 1 mM, lesions became visible after 96 h (Table 1), also limited to the mature source leaves (Figure S2). In general, Spm treatment at 1 mM showed more severe and larger lesions compared to Spd. Moreover, more leaves per plant showed lesions at the same timepoint in Spm-treated plants compared to Spd-treated plants. At lower concentrations, no visible lesions were observed on either Spd- or Spm-treated plants after 120 h. On the basis of these results, we selected 100 µM and 500 µM for subsequent priming experiments.

### 3.2. Polyamines Prime Arabidopsis Resistance Against B. cinerea Infection

Previous data suggested that PA accumulation during *B. cinerea* infection increased plant susceptibility [20], but PA accumulation preceding infection in grapevine reduced susceptibility to *B. cinerea* [28]. This suggested that PAs are effective in promoting subsequent resistance but can be detrimental when accumulating in tandem with the necrotrophic stage of *B. cinerea* infection. Here, we pre-treated Arabidopsis plants with Spd and Spm followed by *B. cinerea* infection 72 h after treatment, ensuring a temporal separation between the presence of PAs and the fungus. After selecting concentrations of PAs that do not elicit cell death phenotypes in the leaves (Table 1), we tested the ability of Spd and Spm at these concentrations to induce resistance against subsequent *B. cinerea* infection.

**Figure 2:**
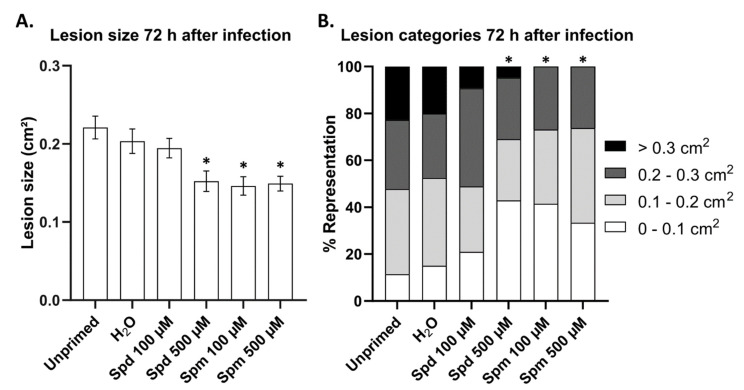
Disease severity of *B. cinerea* infection of Arabidopsis source leaves 72 h after pre-treatment with spermidine (Spd) or spermine (Spm) at 100 or 500 µM. (**A**) Average lesion size of *B. cinerea* infection in plants pre-treated with Spd or Spm at 100 µM or 500 µM compared to untreated and H_2_O-treated controls. Bars represent the mean ± SE of at least 30 biological replicates. Statistical significance is indicated by an asterisk and is based on one-way ANOVA and Dunnett’s multiple comparisons test compared to H_2_O control (* *p* < 0.05). (**B**) Percentage representation of *B. cinerea* lesions categorized according to size. The experiment was repeated three times with consistent results.

Plants pre-treated with both Spm and Spd showed a reduced necrotic lesion development in the leaves of Arabidopsis. We found Spm to be more effective than Spd at the same concentration (Figure 2). At a lower concentration (100 µM), Spd was not able to induce resistance significantly compared to the H_2_O control treatment (Figure 2A). On the contrary, Spm treatment at a lower concentration (100 µM) was similar to the 500 µM dose (Figure 2A). Interestingly, when dividing the lesions into categories according to their size, there were no lesions larger than 0.3 cm^2^ in plants pre-treated with Spm at either concentration (Figure 2B). In comparison, more than 20% of the lesions in control-treated plants were larger than 0.3 cm^2^. Moreover, more than double the lesions of plants treated with Spd (500 µM) and Spm (100 and 500 µM) were smaller than 0.1 cm^2^ compared to the two control treatments (Figure 2B). There seems to be a correlation between the ability of Spm compared to Spd to induce cell death more rapidly (Table 1) and its ability to prime resistance against *B. cinerea*. It was thus clear from our data that when PA accumulation/treatment preceded the presence of *B. cinerea*, subsequent resistance towards *B. cinerea* was induced. The tetraamine Spm was also more effective than the triamine Spd, especially at lower concentrations.

### 3.3. Exogenous Polyamine Treatment Enhanced Endogenous Polyamine Levels

Since it is unclear to what extend the endogenous PA content is affected by exogenous application of PAs, we followed the levels of Put, Spd, and Spm in plants after treatment with Spd or Spm, followed by *B. cinerea* infection. This was important since extracellular PAs can act in different ways:

- they can be imported through PA transporters and contribute to endogenous PA levels;
- they can be broken down in the apoplast through their oxidation [17,53], or they can be directly sensed by thus far unidentified receptors (extracellular signalling).

**Figure 3:**
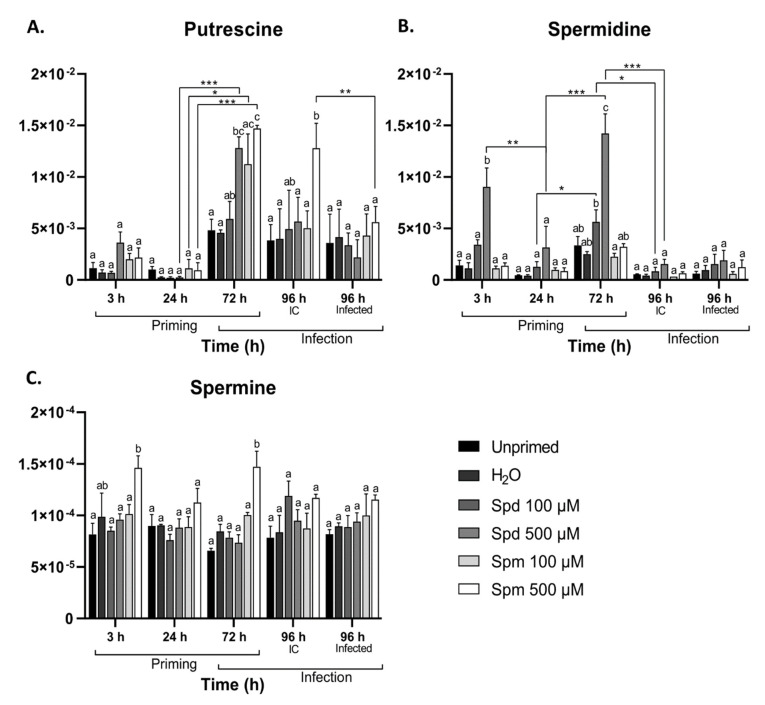
Changes in polyamine content in Arabidopsis source leaves from plants pre-treated with spermidine (Spd) or spermine (Spm) followed by infection with *B. cinerea*. Changes in (**A**) putrescine, (**B**) Spd, and (**C**) Spm in the source leaves of Arabidopsis plants pre-treated with 100 µM or 500 µM Spd or Spm followed by infection with *B. cinerea* or infection control (IC) 96 h later. Values on the *y*-axis have no values, and they represent relative concentrations. Bars represent the mean ± SE of three biological replicates. Statistical significance is indicated by different letters within the same timepoint (*p* < 0.05) and an asterisk (* *p* < 0.05; ** *p* < 0.01; *** *p* < 0.001) between timepoints for the same treatment in terms of two-way ANOVA and Tukey’s multiple comparison test.

Our data indicate that 100 µM exogenous concentrations of Spm and Spd did not significantly increase the endogenous levels of all PAs (3 h data points; Figure 3). On the contrary, treatment with 500 µM Spd and Spm resulted in significant accumulation of Spd and Spm, respectively, at 3 h after treatment (Figure 3B,C), suggesting effective uptake at this enhanced concentration, while Put levels were not significantly affected, although its marginal increase may be explained by PA catabolism of Spm and Spd (Figure 3A). Remarkably, this early wave (for 500 µM Spd and Spm) was followed by a decrease at 24 h and then by a second wave, accumulating at 72 h after treatment where both 500 µM Spd and Spm pre-treatments boosted Put (Figure 3A), while 500 µM Spd pre-treatment boosted Spd (Figure 3B) and 500 µM Spm treatment boosted Spm (Figure 3C). Put levels remained elevated at 96 h (IC) after treatment with 500 µM Spm (Figure 3A). This delayed accumulation was most probably associated with *de novo* synthesis of PAs during the second wave observed at 72 h after treatment. Defoliation greatly repressed Spd levels (Figure 3B), but not the other PAs (Figure 3A,C). Interestingly, we did not observe significant changes in the levels of PAs as a consequence of *B. cinerea* infection, with the exception of a decrease in Put levels in 500 µM Spm pre-treated infected plants. 

### 3.4. Exogenous Polyamines Induced H_2_O_2_ Scavenging Enzymes in Arabidopsis

PAs are known to be broken down in the apoplast through oxidation, releasing H_2_O_2_ as a by-product during each step of deamination [17]. Exogenous application of PAs such as Spd and Spm can thus lead to fluctuations in H_2_O_2_ levels. We therefore studied the effect of exogenous PAs on H_2_O_2_ content and the corresponding H_2_O_2_ scavenging enzymes in response to treatment and *B. cinerea* infection.

**Figure 4:**
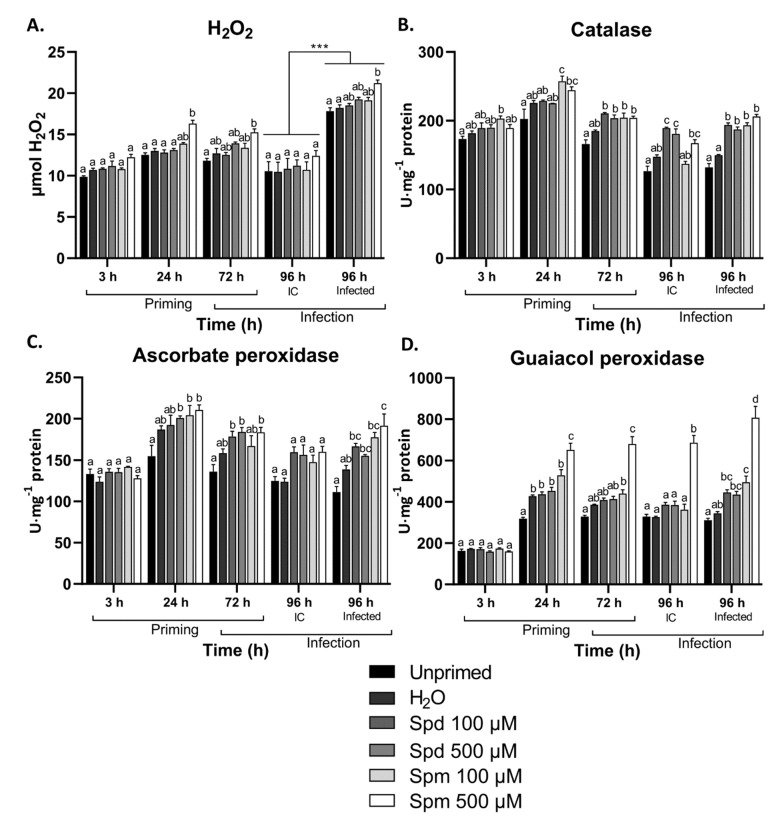
Changes in H_2_O_2_ content and H_2_O_2_ scavenging enzyme activity in Arabidopsis source leaves from plants pre-treated with spermidine (Spd) or spermine (Spm) followed by infection with *B. cinerea*. Changes in (**A**) H_2_O_2_ content and the activity of H_2_O_2_ scavenging enzymes, (**B**) catalase, (**C**) ascorbate peroxidase, and (**D**) guaiacol peroxidase in the source leaves of Arabidopsis plants pre-treated with 100 µM or 500 µM Spd or Spm followed by infection with *B. cinerea* or infection control (IC) 72 h later. Bars represent the mean ± SE of three biological replicates. Statistical significance is indicated by different letters within the same timepoint (*p* < 0.05) and an asterisk (*** *p* < 0.001) between time points for the same treatment in terms of two-way ANOVA and Tukey’s multiple comparison test. A solid line above a timepoint indicates that all the treatments significantly differed between the two timepoints.

We found that only Spm at 500 µM induced a significant accumulation of H_2_O_2_ at 24 h after treatment (Figure 4A), and this correlated remarkably well with the increased overall sugar levels at this timepoint (see below). Similarly, plants pre-treated with 500 µM Spm accumulated higher levels of H_2_O_2_ after 24 h of infection with *B. cinerea* (Figure 4A). Plants treated with a lower concentration of Spm or any of the concentrations of Spd did not show significant changes in H_2_O_2_ after treatment or infection (Figure 4A). Infection itself greatly boosted H_2_O_2_ increases (Figure 4A). The activities of catalase (CAT), ascorbate peroxidase (APX), and guaiacol peroxidase (GPX) showed a general higher trend in all plants pre-treated with PAs (Figure 4B–D). The activity of guaiacol peroxidase significantly increased from 24 h onwards in the leaves of plants pre-treated with 500 µM Spm (Figure 4D). After leaf defoliation, plants treated with Spd and Spm also showed significantly higher activities for CAT and APX compared to the control treatments (Figure 4B,C). After infection, CAT activity was significantly higher in all the PA-treated plants compared to the controls, although a rather similar pattern was also observed in uninfected controls (Figure 4B). The activity of APX was only significantly higher after infection in plants treated with 500 µM Spm, whereas both concentrations of Spm showed significantly higher levels of GPX activity after infection.

### 3.5. Polyamines Induce Temporal Sugar Accumulation after Treatment and B. cinerea Infection

With sugars forming an integral part of plant metabolism and defence strategies during stress conditions, they function as important translators of the plants’ energy status [54,55,56]. These findings prompted us to study the effect of exogenous PAs on soluble sugar content both after treatment and in response to *B. cinerea* infection.

**Figure 5:**
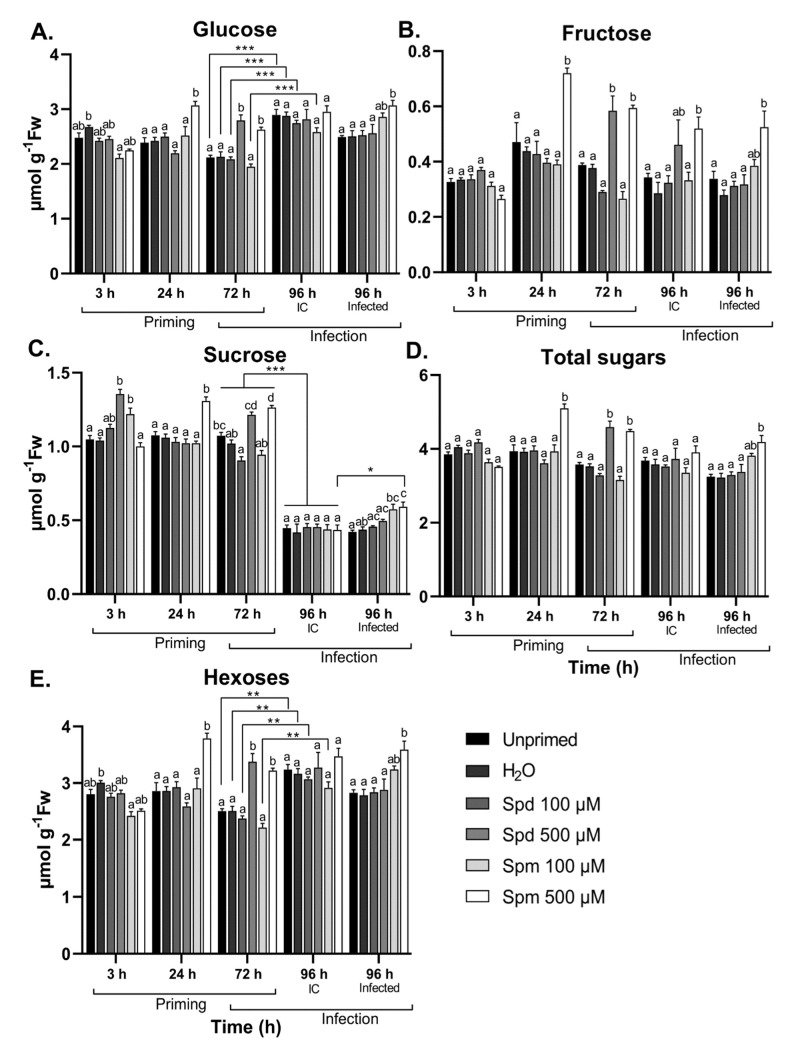
Effect of spermidine (Spd) or spermine (Spm) pre-treatment followed by *B. cinerea* infection on soluble sugar content in the leaves of Arabidopsis plants. Changes in (**A**) glucose, (**B**) fructose, (**C**) sucrose, (**D**) total sugars (glucose, fructose, and sucrose), and (**E**) hexoses (glucose and fructose) in the source leaves of Arabidopsis plants pre-treated with 100 µM or 500 µM Spd or Spm followed by infection with *B. cinerea* or infection control (IC) 72 h later. Bars are the mean ± SE of six biological replicates. Statistical significance is indicated by different letters within the same timepoint (*p* < 0.05) and with an asterisk (* *p* < 0.05; ** *p* < 0.01; *** *p* < 0.001) between time points for the same treatment in terms of two-way ANOVA and Tukey’s multiple comparison test. A solid line above a timepoint indicates that all the treatments significantly differed between the two timepoints.

Plants treated with 500 µM Spm showed significant increases in soluble sugars (glucose (Glc), fructose (Fru) and sucrose (Suc)) 24 h after treatment when compared to the controls (Figure 5A–C). Additionally, 72 h after treatment with either Spd or Spm at 500 µM, plants accumulated significant amounts of Glc, Fru, and Suc compared to the control treatments. This was also evident for the total hexose (Glc + Fru) (Figure 5D) and total soluble sugar levels (Glc + Fru + Suc) (Figure 5E), suggesting that a general increase in sugar content occurs after PA treatment at higher concentrations. The level of Suc greatly decreased because of leaf defoliation, when comparing 72 h samples, 96 h IC, and 96 h infected plants (Figure 5C). In contrast, leaf defoliation resulted in significant accumulation of hexoses (Figure 5E). Thus, leaf defoliation decreased Suc to hexose ratios to a great extent. After infection, however, only plants pre-treated with 500 µM Spm showed significantly higher levels of Glc, Fru, and Suc compared to the two controls, while the 100 µM Spm treatment also increased but did not reach significant levels (Figure 5A–C). Plants pre-treated with 500 µM Spm also accumulated Suc to levels significantly higher than IC control plants with the same treatment. Neither Glc nor Fru accumulated to higher levels in infected compared to IC in treated plants, suggesting that Spm specifically promoted Suc accumulation after infection (Figure 5A–C). Treatment with Spd did not influence soluble sugar content during *B. cinerea* infection. There is a clear correlation between elevated PAs (Figure 3) and overall sugar levels (Figure 5) at the 72 h after priming timepoint. This also correlated with the ability of Spm, but not Spd, to prime the accumulation of H_2_O_2_ after *B. cinerea* infection. 

### 3.6. Polyamines Differentially Modulated Nitrate Reductase Activity in Arabidopsis Leaves

**Figure 6:**
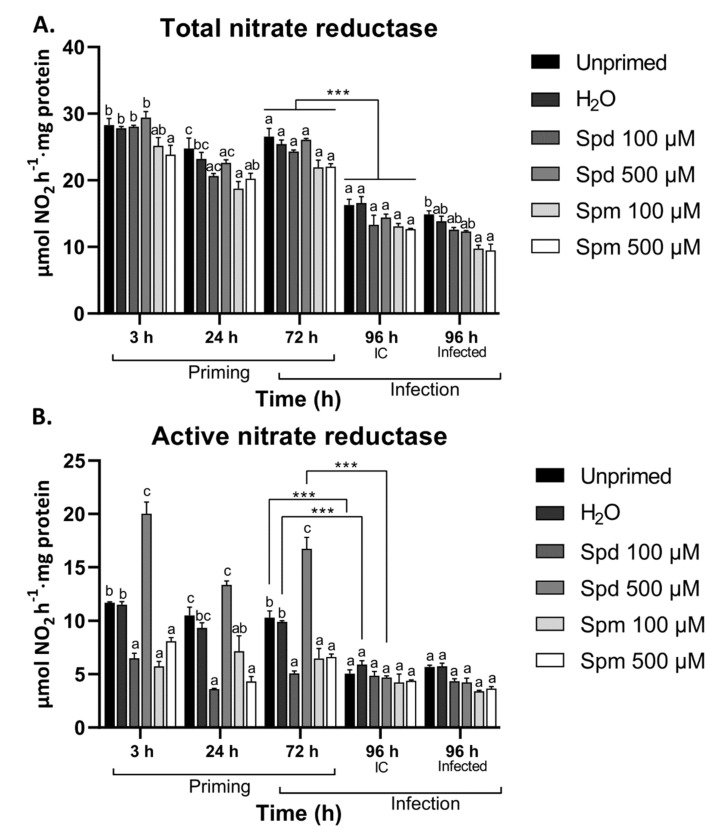
Effect of exogenous spermidine (Spd) or spermine (Spm) pre-treatment followed by *B. cinerea* infection on nitrate reductase (NR) activity in the source leaves of Arabidopsis plants. Changes in the (**A**) total NR activity (active and inactive) and the (**B**) active NR (unphosphorylated) in the source leaves of Arabidopsis plants pre-treated with 100 µM or 500 µM Spd or Spm followed by infection with infection control (IC) or *B. cinerea* 72 h later. Bars are the mean ± SE of three biological replicates. Statistical significance is indicated by different letters within the same timepoint (*p* < 0.05) and an asterisk (*** *p* < 0.001) between timepoints for the same treatment in terms of two-way ANOVA and Tukey’s multiple comparison test. A solid line above a timepoint indicates that all the treatments significantly differed between the two timepoints.

Polyamines and nitrogen (N) metabolism interplay to connect PA and N metabolism to carbon fixation and secondary metabolism [57]. During abiotic and biotic stress, plants remobilize C and N into signalling molecules such as PAs, GABA, and proline. These signalling molecules in turn function to regulate the N assimilation/partitioning, and this process largely depends on the C status. On the other hand, NR is also extremely sensitive to the plants’ energy status and stress conditions [58,59]. Interestingly, the NR activity in wheat leaf segments was shown to be differentially regulated in a time-dependent manner in response to PA treatment [36]. However, to our knowledge, the effect of exogenous PA treatment on NR activity has not been explored. Here, we explored both the total NR (active and inactive forms) and the active NR activity to establish whether exogenous PAs affect the accumulation of NR protein or the activation state of the protein (Figure 6).

The total NR activity was significantly lower at 3 h after treatment with 500 µM Spm and at 24 h after treatment with 100 µM Spm compared to the control treatments (Figure 6A). This suggests a time/dose-dependent regulation of NR protein accumulation. Treatment with Spd at both concentrations did not have any significant effect on the total NR activity. Active NR on the other hand showed some peculiar responses. Treatment with Spm at both concentrations and treatment with 100 µM Spd inhibited the active NR activity whereas treatment with 500 µM Spd induced the active NR (Figure 6B). This effect remained prominent until 72 h after treatment. Leaf defoliation resulted in reduced total NR activity irrespective of the treatment (Figure 6A). Active NR activity did not show a significant reduction after defoliation in plants pre-treated with Spm at either concentration or Spd at 100 µM since they were already at comparable levels 72 h after treatment, while a decrease was observed for the other treatments (Figure 6B). Total NR activity was significantly lower in plants treated with Spm after infection compared to the control treatments (Figure 6A). The active NR activity did not significantly change after *B. cinerea* infection for any of the treatments (Figure 6B). Moreover, Spd-treated plants did not show any clear differences in response to infection when compared to the non-infected controls (Figure 6B).

Our data indicate that exogenous Spm reduced both the total and active NR activity in Arabidopsis. On the other hand, Spd seemed to regulate active NR activity in a concentration-dependent manner. These data seem to be in contrast with the effect of PAs on soluble sugars. This points to potential changes in the ratio of N:C after PA treatment. It also suggests that the level of NR protein was less affected by PAs as compared to the activation state of the enzyme.

### 3.7. Exogenous Spm But Not Spd Induce GABA Accumulation after Treatment and B. cinerea Infection

The non-proteinogenic amino acid GABA is an important metabolic component and signalling entity during abiotic and biotic stresses [27]. During necrotrophic infection, GABA serves as substrate to fuel the TCA cycle, which is a key energy producing pathway for metabolic processes required for plant defences [60]. Components produced by the TCA cycle also form part of the plants defence arsenal. In addition, GABA is believed to act as an effective signalling molecule, translating stress signals between cells [61]. As apoplastic PAs can be converted to GABA through oxidation, GABA can be an important component in PA-induced immune responses. We thus measured the endogenous GABA content and its precursor (glutamate (Glu)) in Arabidopsis leaves in response to treatment with Spd and Spm followed by *B. cinerea* infection.

**Figure 7:**
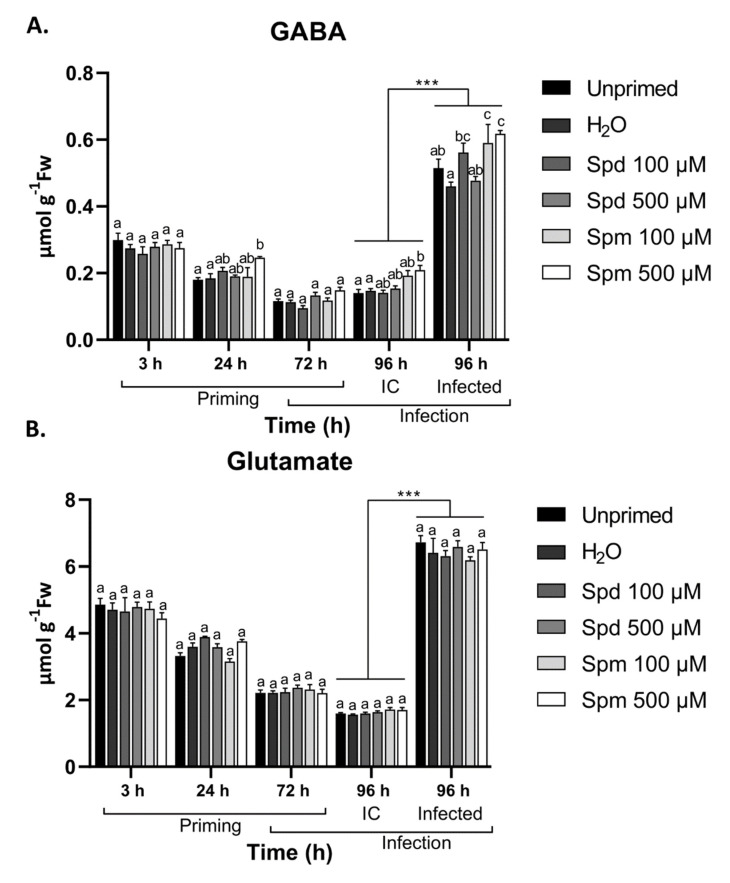
Effect of spermidine (Spd) or spermine (Spm) pre-treatment and *B. cinerea* infection on γ-aminobutyric acid (GABA) and glutamate content in the leaves of Arabidopsis plants. Changes in (**A**) GABA and (**B**) glutamate in the source leaves of Arabidopsis plants pre-treated with 100 µM or 500 µM Spd or Spm followed by *B. cinerea* infection or infection control (IC) 72 h later. Bars represent the mean ± SE of three biological replicates. Statistical significance is indicated by different letters within the same timepoint (*p* < 0.05) and with an asterisk (*** *p* < 0.001) between time points for the same treatment in terms of two-way ANOVA and Tukey’s multiple comparison test. A solid line above a timepoint indicates that all the treatments significantly differed between the two timepoints.

Plants treated with 500 µM Spm showed a significant increase in GABA levels 24 h after treatment compared to control treatments (Figure 7A). This was not the case for any of the other treatments over the first 72 h time period. Leaf defoliation again induced significant GABA accumulation in plants pre-treated with 500 µM Spm, and to a lesser extent in plants treated with 100 µM Spm (Figure 7A). The levels of Glu were not significantly altered throughout the first 72 h for any of the treatments (Figure 7B), suggesting that GABA accumulation likely occurred through PA oxidation rather than through Glu-dependent pathways. Infection caused a very strong increase of GABA and Glu, irrespective of the treatment (Figure 7A,B). However, plants pre-treated with both concentrations of Spm showed significantly higher levels of GABA when compared to the control treatments (Figure 7A). Treatments with Spd did not show any significant changes in GABA levels after infection with *B. cinerea* (Figure 7A).

Our data suggest that GABA levels were (slightly) differentially regulated by exogenous Spm and Spd. Of particular interest was the accumulation of GABA after a subsequent stress such as mechanical damage (defoliation) or infection with *B. cinerea*. This seemed to be also the case for soluble sugars (Figure 5), especially after infection where Glc, Fru, and Suc accumulated only in plants pre-treated with Spm. In addition, H_2_O_2_ also accumulated more profoundly in Spm-treated plants after infection (Figure 4), potentially pointing to interplay between Spm, sugar, and H_2_O_2_ signalling events. 

### 3.8. Defence-Related Amino Acids Accumulate in Spm-Treated Plants after Infection

During abiotic or biotic stress, several defence responses in plants involve the readjustment of amino acid metabolism to serve as precursors for defence compounds or to counteract the detrimental impact directly [38,62]. Amino acids are also important nitrogen sources and can be transported from healthy tissue to infected parts to supply the plants with the required resources for defence strategies. Besides the effect of PAs on GABA and Glu, we also explored the effect of PA treatment on a range of additional amino acids after treatment and infection.

**Figure 8:**
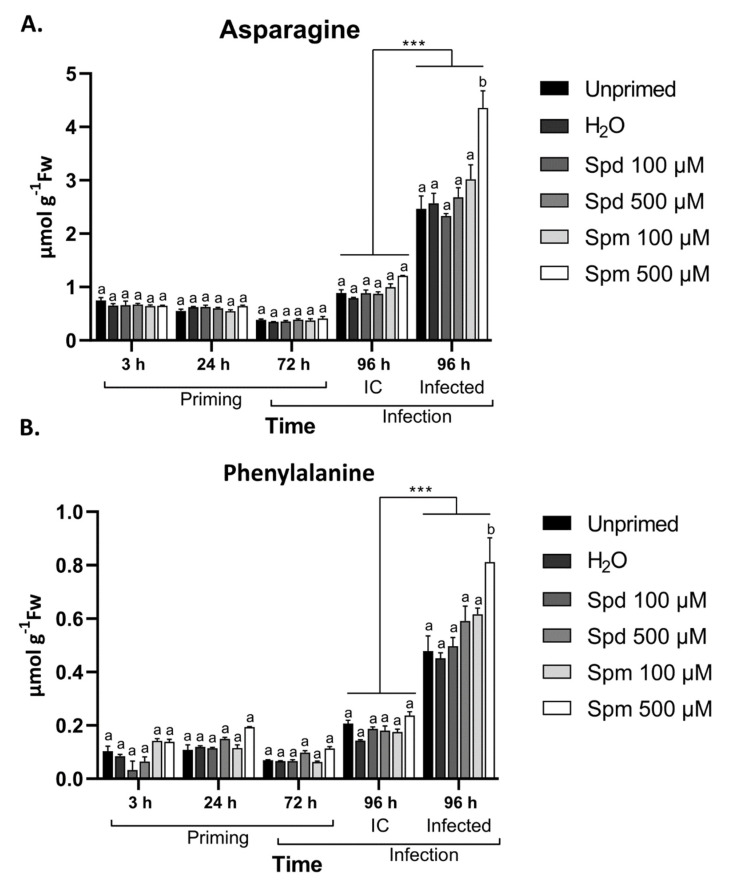
Spermine (Spm) pre-treatment induced asparagine and phenylalanine accumulation in the leaves of Arabidopsis plants after *B. cinerea* infection. Changes in (**A**) asparagine and (**B**) phenylalanine in the source leaves of Arabidopsis plants pre-treated with 100 µM or 500 µM spermidine (Spd) or Spm followed by *B. cinerea* infection 72 h later. Infection control (IC) represents uninfected plants. Bars are the mean ± SE of three biological replicates. Statistical significance is indicated by different letters within the same timepoint (*p* < 0.05) and with an asterisk (*** *p* < 0.001) between time points in terms of two-way ANOVA followed by Tukey’s multiple comparison test. A solid line above a timepoint indicates that all the treatments significantly differed between the two timepoints.

To gain a better understanding of the changes in amino acid levels in response to PA treatment, we analyzed 19 different amino acids over the course of treatment and infection. Although all amino acids accumulated in response to infection with *B. cinerea* (data not shown), asparagine (Asn) (Figure 8A), the amino acid with the highest ratio of N:C, and phenylalanine (Phe) (Figure 8B), a major contributor to the synthesis of phenolic compounds, increased most strongly after infection, specifically caused by infection (Figure 8A,B). Among infected plants, those treated with 500 µM Spm stood out (Figure 8A,B). Overall, correlations were detected between soluble sugars (Figure 5), H_2_O_2_ levels (Figure 4), and the amino acids GABA, Asn, and Phe (Figure 8) after *B. cinerea* infection in plants treated with 500 µM Spm but not with 500 µM Spd. Taken together, this indicates that Spm pre-treatment is accompanied by major metabolic shifts after infection with *B. cinerea*. 

## 4. Discussion

PAs, the small aliphatic polycations present in eukaryotic organisms, function in several key physiological and developmental processes and stress responses in plants [13,63]. Their biosynthesis and degradation are also highly responsive to environmental conditions [5,6,7]. There are several studies indicating that the major PAs shape plant responses to abiotic and biotic stresses [5,6,10,31,64]. However, there is emerging evidence that Spm has a unique role in the induction of defence responses, one that is not shared by the other PAs [4]. For instance, it was shown that Spm is the only PA able to suppress multiplication of cucumber mosaic virus in Arabidopsis [23].

We showed that both Spd and Spm were able to induce cell death in the source leaves of Arabidopsis at higher concentrations. However, Spm showed faster and more severe lesion development compared to Spd from 48 h to 120 h after treatment (Table 1). The induced cell death could be explained by the release of H_2_O_2_ from the oxidation of Spd/Spm by either PAO or DAO enzymes, and this appeared to be a slow and steady process evolving between 48 and 120 h (Table 1). It was also shown that tobacco mosaic virus (TMV)-induced hypersensitive response (HR) required the activity of PAO [49,65]. The authors also showed that this PAO had a 10 times higher affinity for Spm over Spd, which might explain why Spm showed faster and more severe lesion development. Similarly, Spm was more effective in inducing defence-related gene expression in Arabidopsis [23]. Additionally, recent findings showed that exogenous Spm induced the accumulation of phosphatidic acid, known to induce cell death [26].

It is clear that Spm, compared to Spd, is more effective in priming resistance against *B. cinerea* in Arabidopsis plants (Figure 2). This was particularly clear at concentrations of 100 µM, where Spd lost its ability to induce subsequent resistance. Previous studies showed that tomato plants over-accumulating PAs were more susceptible to *B. cinerea* infection due to the attenuation of ethylene defence responses [20]. In contrast, grapevine plants in which PA accumulation was induced through drought stress showed lower susceptibility to subsequent *B. cinerea* infection [28]. By using pharmacological inhibitors, the authors demonstrated that the oxidation of PAs through PAOs or DAOs, scored after several days, correlated well with subsequent resistance against *B. cinerea*. Additionally, H_2_O_2_ derived from PA oxidation is also known to strengthen the cell wall during (a)biotic stresses [18,66]. Theoretically, compared to Spd, Spm can generate twice the amount of H_2_O_2_ during their complete oxidation to Put. Thus, it seems possible that Spm treatment produces more H_2_O_2_, leading to a stronger activation of defence responses, perhaps through H_2_O_2_ signalling. However, the very low concentrations of PAs used in this study, as compared to these previous studies, raises the question as to whether substantial amounts of Put and H_2_O_2_ can be generated for this purpose. Likely, this is not possible within the shorter timeframe of about 24 h in which the initiation of priming effects are generally believed to occur. Accordingly, no significant H_2_O_2_ and Put/Spd increases were detected after 3 h in Spm-treated plants (Figure 3 and Figure 4). Moreover, the H_2_O_2_ peak observed after 24 h in 500 µM Spm-treated plants did not correlate with increased Put/Spd levels at that time point (Figure 3 and Figure 4), on the contrary. Likely, this H_2_O_2_ peak after 24h had another origin (see below). We conclude that apoplastic oxidation of PAs was most probably not a major factor involved in short-term priming effects (24 h) mediated by low concentrations of PAs, discriminating these effects from treatments using higher concentrations and requiring more time, also contributing to defence but being different from genuine priming effects from the one described here. Our data show that Spm, and to a lesser extent Spd, were able to prime longer-term resistance against *B. cinerea* at lower concentrations than those used in a recent contribution [21]. During this priming effect, Spm, H_2_O_2_, or GABA signalling processes may come into play. Although a PA sensor has been described in bacteria, no such sensor has been discovered yet in plants [25].

Remarkably, exogenous treatment with 500 µM Spd and Spm resulted in a two-phase endogenous accumulation of PAs. A short-term accumulation occurred at 3 h after treatment, followed by a second wave at 72 h after treatment (Figure 3). The initial accumulation was probably a result of cellular import, whereas the second wave at 72 h points to overall *de novo* PA synthesis, supported by the simultaneous accumulation of Put together with Spd or Spm. The unexpected repression of endogenous PA levels at 24 h was rather puzzling (Figure 3). Interestingly, focusing on Put (Figure 3A), the decrease between 3 h and 24 h of treatment was stronger in the PA-treated plants as compared to the controls. This hints at a regulated temporal breakdown of intracellular Put, perhaps with the purpose of using the gained C to prioritise the synthesis of other compounds. One of such possible compounds are the sugars, Suc in particular. Accordingly, we detected early Suc accumulation (Figure 5C), in agreement with what we found before for GABA priming [30]. A previous study already demonstrated that Suc signalling pathways are connected to PA metabolism and/or signalling events [31]. Exogenous Suc induced the accumulation of Put, Spd, and Spm in Arabidopsis plants to induce atrazine resistance. Therefore, it seems possible that Suc signalling events are responsible for the strong second wave of PAs observed at 72 h, perhaps in concert with specific intracellular PA signalling events through feed-forward PA synthesis [24,67]. Another interpretation may be that the uptake of PAs at higher concentrations during exogenous treatments [53] may result in fluctuations of the C:N ratio, activating mechanisms to restore the C:N balance, inducing the synthesis of Suc. PAs are known to be involved in C:N signalling events in plants [33,34]. Thus, for the moment, it is hard to judge whether intracellular PA signalling precedes Suc signalling or the other way around, and further studies taking into account more timepoints are required. The clear biological effect of 100 µM Spm on disease tolerance (Figure 2) can probably not be explained by cellular uptake (Figure 3) and a cascade of potential intracellular signalling events, as described above for the 500 µM treatments. We hypothesise that these effects mostly rely on extracellular PA signalling events independent of PA uptake, similar to the different modes of action that we recently proposed for different concentrations of GABA during priming [30].

We were able to visualise the increased amounts of H_2_O_2_ after the 500 µM exogenous Spm treatments. The increase in H_2_O_2_ at the 24 h and 72 h timepoints likely points to *de novo* production of H_2_O_2_ that is not associated with extracellular PA oxidation (see above). A more likely explanation is that this H_2_O_2_ is potentially through feed-forward activation of NADPH-xidases, as previously explained [57]. The use of NADPH-oxidase or PAO inhibitors or mutants should be considered in future studies to confirm the exact source of H_2_O_2_. In addition, H_2_O_2_ significantly accumulated after *B. cinerea* infection in plants treated with Spm but not with Spd (Figure 4). This points at a possible priming of NADPH-oxidase responses after perception of *B. cinerea*. There are some indications that apoplastic PAOs and NADPH-oxidases act in a feed-forward loop to activate defence responses in plants [57]. However, as neither Spm at lower concentration nor Spd treatments showed a similar accumulation of H_2_O_2_ but still induced resistance, it is still possible that a ROS burst during infection is not the most crucial factor in PA-induced immunity. It is also interesting that although there was no clear induction of H_2_O_2_ for Spd, H_2_O_2_ scavenging enzymes were still strongly induced after treatment and infection in these plants. The effect of PAs on ROS and ROS scavenging enzymes seems to be acting as a double-edged sword in plants [68]. Depending on the environmental conditions and the localization of PAs, ROS accumulation or ROS scavenging can be promoted. We found that H_2_O_2_ scavenging enzymes were promoted in plants pre-treated with PAs (Figure 4), although this was not associated with a clear decrease in H_2_O_2_ content, suggesting that ROS scavenging systems are responding to H_2_O_2_ produced by PA oxidation. It is also interesting that activation of H_2_O_2_ scavenging enzymes were more profound in plants treated with PAs after *B. cinerea* infection, confirming that Spm acts as a genuine priming compound.

Treatment with either of the PAs resulted in significant soluble sugar accumulation (Figure 5). On the contrary, only Spm-treated plants accumulated higher levels of soluble sugars in response to infection when compared to the control treatments. Soluble sugars such as Glc and Suc are known for their role in stress signalling events and serving as substrates for the synthesis of defence compounds [54,69,70]. In mature leaves such as the ones studied here, exogenous PAs stimulated accumulation of small soluble sugars, which have no inhibitory effect on sucrose non-fermenting related kinase 1 (SnRK1) activity in mature leaves [71]. Moreover, in general, most biotic stresses further stimulate SnRK1 activity [72], associated with Suc-specific signalling pathways [69,70]. The increased sugar levels after *B. cinerea* infection in Spm-treated plants are expected to fuel the *de novo* synthesis of GABA, Asn, and Phe, accumulating to a considerable extent. The accumulation of PAs and enhanced SnRK1 activities have been linked before to catabolic processes under stress, suggesting that increased starch degradation may be one of the driving forces for small soluble sugar accumulation [33,34]. The increase in soluble sugars in plants treated with 500 µM Spm after infection (Figure 5) further supports a role for Spm to prime longer-term resistance in plants. The substantial decrease in Suc/hexose ratio after leaf defoliation may be associated with altered invertase or sucrose phosphate synthase (SPS) activities, but this requires further investigation and at the same time urges further research on how the sugar signalling context may differ when infections would occur on attached leaves. It can be hypothesised that defoliation-associated wounding may signal “danger”, potentially leading to enhanced SnRK1 activities, inhibiting SPS activity. This is also supported by our NR activity assays showing reduced active NR activity after leaf defoliation (Figure 6), since SnRK1 regulates both SPS and NR in a negative way [58,59].

NR activity is a key contributor of nitrogen for amino acid synthesis in plants. However, NR is also responsible for the production of nitric oxide (NO), a key signalling molecule in plants [37]. It is interesting, however, that both the total and active NR activity were inhibited by Spm (Figure 6). In contrast, Spd had no effect on the total NR activity, but significantly induced the active NR activity. The inhibition of NR activity is typically associated with stress conditions, for instance regulated by Ca^2+^-dependent protein kinases and SnRK1 [59,73], suggesting that SnRK1 would be active under these conditions, inhibiting both the expression and activity of NR. Such a scenario would explain why Spm showed significant inhibition of NR. However, in a study performed in wheat leaf segments, it was shown that both Spd and Spm can inhibit NR activity in the short term but stimulate activity over longer periods [36]. They showed that NO was involved in the regulation of NR activity by PAs. However, it is difficult to compare our data with these findings since they did not discriminate between the active and the total NR protein and their samples most probably represent a combination thereof. Additionally, yet another study showed that Spd and Spm inhibit the induction of NR activity in cotyledons of radish [74]. It was also shown that both Spd and Spm can bind to 14-3-3 proteins to prevent their interaction and inhibition of NR activity [35]. For the moment, it is quite complex to understand at which level NR activity is regulated by exogenous PA treatments, since PAs can be broken down in the apoplast [66], but also transported across the membrane [26]. Moreover, as suggested before for GABA [30], it cannot be excluded that extracellular PAs may be influencing SnRK1 in an opposite way as compared to intracellular PAs, further complicating things.

Several plant defence responses involve the readjustment of amino acid metabolism to either counteract the detrimental effects directly, or provide the precursors for defence compounds [75]. Amino acids such as GABA and Pro typically accumulate in response to abiotic and biotic stresses and directly or indirectly alleviate these stresses. On the other hand, amino acids such as Phe, Asn, and Trp serve as the precursors for defence compounds such as secondary metabolites [76]. We found significant accumulation of GABA after treatment with Spm (Figure 7), followed by a second wave after infection with *B. cinerea*. The protective role of GABA during *B. cinerea* infection is well described in plants, and it involves the activation and fuelling of the TCA cycle under oxidative stress and promoting pro-survival strategies [60,77]. It is possible that GABA is directly produced from PAs after exogenous application. However, only Spm-induced GABA levels and Spm-treated plants accumulated higher levels after infection. This rather points into the direction of *de novo* synthesis of GABA, independent of PA oxidation. In fact, Spm induced membrane depolarization in the roots of pea plants, releasing Ca^2+^, which is the main regulator of *de novo* GABA synthesis through glutamate decarboxylase [78]. It is also possible that exogenous Spm is more rapidly taken up into the cell compared to Spd, due to higher affinity of PA transporters for Spm [53], resulting in intracellular oxidation of Spm to produce Put, and from there GABA. It is also possible that small amounts of GABA produced by PA oxidation might promote endogenous GABA synthesis through a feed-forward approach [79].

Additionally, we found that Phe and Asn significantly accumulated after *B. cinerea* infection in plants pre-treated with Spm (Figure 8). These amino acids were previously associated with resistance against *B. cinerea*. In Arabidopsis, tomato, petunia, and *Chrysanthenum* plants over-accumulating or treated with Phe, several phenylpropanoids associated with the inhibition of *B. cinerea* germination and growth accumulated to induce resistance against this fungus [80,81]. Additionally, the same tomato abscisic acid -deficient plants showing over-activation of the GABA-shunt and GABA accumulation with enhanced resistance against *B. cinerea* also showed significant accumulation of Asn [82]. In the wild-type plants, Asn levels were severely depleted, whereas mutants showed continuous accumulation of Asn. It is interesting that a previous study found that sugar depletion also caused depletion of Asn levels [83]. It was proposed that *B. cinerea* consumption of apoplastic sugars in susceptible plants could be responsible for Asn depletion [82]. Our data also support these findings, as we found a correlation between soluble sugar accumulation and Asn levels in plants treated with Spm after *B. cinerea* infection (Figure 5 and Figure 8).

## 5. Conclusions

We showed for the first time that below-millimolar concentrations of exogenous Spd and Spm were effective in inducing longer-term resistance to *B. cinerea* through a genuine priming mechanism. Our data support the literature that PAs are central regulators of plant metabolism and ROS dynamics, especially during infection with *B. cinerea*. PA treatment also promoted endogenous PA synthesis over longer periods. We detected discrepancies between the effect of exogenous Spd and Spm on these parameters. Only Spm was able to affect H_2_O_2_ levels after treatment and infection, however, both Spd and Spm promoted H_2_O_2_ scavenging enzyme activity, urging further research into the origin of this H_2_O_2_. Additionally, soluble sugars significantly accumulated in plants treated with either Spd or Spm. Although the exact reason for sugar accumulation after exogenous PA treatment is still unclear, emerging insights suggest a role for communication between PAs and sugar signalling pathways (possibly through SnRK1), warranting further studies. Interestingly, NR activity, a main target of SnRK1, was inhibited by Spm and low concentrations of Spd but enhanced by higher levels of Spd. It was also evident that amino acid levels were only affected by Spm treatments and not by Spd. Treatments with Spm induced GABA accumulation after treatment, and showing higher levels of GABA, Phe, and Asn after *B. cinerea* infection.

It is important for future research to uncover the exact mechanisms that translate exogenous PA treatments to intracellular sugar, N, and amino acid metabolism. Our data hint at possible extracellular PA signalling at 100 µM and intracellular signalling at 500 µM, the latter probably in heavy cross-talk with Suc signalling. The hunt is open to uncover possible PA sensors in plants, encouraged by their existence in bacteria and by the observations in this work. Perhaps extracellular PA sensing is intrinsically linked to lipid signalling now recognized as a crucial component of PA signalling events [84].

There is an urgent need for natural and sustainable alternatives for toxic agrochemicals. The results presented here clearly indicate that Spm, at a very low dose, has great potential as a cheap and environmentally friendly priming compound to be further tested in an array of crops, potentially acting against a broader spectrum of pathogens. It will be interesting to explore synergistic effects in combination with other types of promising priming compounds such as fructans [35,42]. Such formulations may not only turn out to be effective against an array of biotic stresses, but they may also lead to cross-tolerance and substantially alleviate the impact of abiotic stresses [81].

## Figures and Tables

**Table 1:** Number of leaves per plant showing necrotic lesion development after treatment with different concentrations of spermidine or spermine. Control plants were treated with H_2_O control. Values are the mean ± SD of five plants.

Treatment	48 h	96 h	120 h
Control	-	-	-
Spermidine 100 µM	-	-	-
Spermidine 500 µM	-	-	-
Spermidine 1 mM	-	2.75 ± 0.57	4.5 ± 0.57
Spermine 100 µM	-	-	-
Spermine 500 µM	-	-	-
Spermine 1 mM	3 ± 0.70	5.2 ± 0.47	6.8 ± 1.09

## Data Availability

Data will be made available by the authors upon request.

## Supplementary Materials

The following are available online at https://www.mdpi.com/2218-273X/11/2/223/s1: Figure S1: Photograph illustrating *B. cinerea* lesion development 72 h after infecting Arabidopsis source leaves. Figure S2: Photographs of necrotic lesion development in response to spermine and spermidine treatments at different concentrations after 120 h.
